# COVID-19 in Pregnancy—Perinatal Outcomes and Vertical Transmission Preventative Strategies, When Considering More Transmissible SARS-CoV-2 Variants

**DOI:** 10.3390/jcm10163724

**Published:** 2021-08-21

**Authors:** Marcin Januszewski, Laura Ziuzia-Januszewska, Malgorzata Santor-Zaczynska, Alicja A. Jakimiuk, Tomasz Oleksik, Marek Pokulniewicz, Kamil Pluta, Waldemar Wierzba, Artur J. Jakimiuk

**Affiliations:** 1 Department of Obstetrics and Gynecology, Central Clinical Hospital of the Ministry of Interior and Administration, 02-507 Warsaw, Poland; lek.med.mjanuszewski@gmail.com (M.J.); msanib@wp.pl (M.S.-Z.); tomek.oleksik@gmail.com (T.O.); marek.poko@gmail.com (M.P.); kamil_pluta@wp.pl (K.P.); waldemar.wierzba@cskmswia.gov.pl (W.W.); 2 Department of Otolaryngology, Central Clinical Hospital of the Ministry of Interior and Administration, 02-507 Warsaw, Poland; lauraziuzia@gmail.com; 3 Department of Plastic Surgery, Central Clinical Hospital of the Ministry of Interior and Administration, 02-507 Warsaw, Poland; alajakimiuk@hotmail.com; 4 Satellite Campus in Warsaw, University of Humanities and Economics, 01-513 Warsaw, Poland; 5 Center for Reproductive Health, Institute of Mother and Child, 01-211 Warsaw, Poland

**Keywords:** COVID-19, pregnancy, vertical transmission, SARS-CoV-2, new variants, perinatal outcomes

## Abstract

The COVID-19 pandemic affected the physical and mental health of people around the world and left unprepared health care systems struggling to mount an adequate response. Understanding the impact of COVID-19 on pregnancy in terms of perinatal and fetal outcomes is essential to propose strategies for mminimising viral transmission. Overall, 91 pregnant women in labour, or with indication for induction of labour, with COVID-19 were admitted to hospital. On the day of admission, each pregnant woman underwent a nasopharyngeal swab to validate SARS-CoV-2 infection. Whenever delivery was by caesarean section, an amniotic fluid sample was collected after uterus incision. Neonates were tested twice: first by nasopharyngeal swab at birth and secondly either at 24 h after (when babies were isolated) or at discharge (when rooming-in). All samples underwent rRT-PCR testing for SARS-CoV-2. The SARS-CoV-2 RNA tests by nasopharyngeal swab of the pregnant women produced positive results in 47 patients. This cohort gave birth to 48 infants who were double tested by nasopharyngeal swab and included in the prospective observational study. Moreover, in this same cohort, 39 amniotic fluid samples were taken during caesarean section. All samples underwent rRT-PCR testing for SARS-CoV-2 and came back negative. The study results suggest a low risk of vertical transmission of COVID-19 and favourable perinatal outcomes due to adequate preventative strategies. This approach may prove to be more beneficial in the new SARS-CoV-2 variants era.

## 1. Introduction

Coronavirus disease 2019 (COVID-19) is an infection caused by the severe acute respiratory syndrome coronavirus 2 (SARS-CoV-2) and characterized as a pandemic by the World Health Organization on 11 March 2020. Like SARS-CoV-1 and MERS, it is a single-stranded RNA virus belonging to the Coronaviridae family [1] and primarily transmitted through respiratory droplets (droplet particles being >5–10 μm in diameter). It can also be transmitted via surfaces contaminated with the secretions of an infected person and by airborne routes (<5 μm in diameter droplet nuclei) [2]. SARS-CoV-2 is present in bronchoalveolar lavage fluid, sputum, nasal and pharyngeal swabs, faeces, blood, and urine [3]. Its less severe course of illness and lower fatality rate (of approximately 2%) [4] than severe acute respiratory syndrome (SARS) (9.5%) [4] and Middle East respiratory syndrome (MERS) (35%) [5], combined with its high infectivity, has enabled the pathogen to spread worldwide.

The fact that SARS-CoV-2 has the potential to spread to different extrapulmonary tissues, based on the mechanisms of infection and virus tropism to various determinants, has an impact on the clinical outcomes [6]. The first step of SARS-CoV-2 infection is recognition of the human receptor angiotensin I-converting enzyme 2 (ACE2) on the surface of host cells. This phase is mediated by the viral spike (S) protein which is essential for its virulence. Secondly, activation of the S protein takes place. Fusion of the viral and cellular membranes is mediated by transmembrane serine, protease 2 [7,8,9,10,11]. The TMPRSS2 gene is regulated by androgens, which may explain the higher susceptibility of men to severe forms of COVID-19 [12].

Vertical transmission is defined as the passage of infectious diseases or pathogens from one generation to another. It includes transmission in utero during the antepartum period, the intrapartum period by exposure to blood and secretions, and the postpartum period via breastfeeding and direct contact. According to the classification system in Shah et al., [13] a confirmed case of congenital infection is defined as detection of the virus in the neonate of a mother with SARS-CoV-2 infection by PCR analysis of umbilical cord blood within the first 12 h of birth, or in amniotic fluid collected prior to rupture of membrane, or in the case of a caesarean section, in a sample taken before the rupture of the membranes. The classification of a probable case includes detection of the virus in the neonate by PCR analysis of a nasopharyngeal swab sample at birth (collected after cleaning the baby), or by a placental swab from the foetal side of the placenta in a neonate born via caesarean section before rupture of the membrane or placental tissue. When there are no clinical features of infection in the new-born and mother with SARS-CoV-2 infection, confirmation can be achieved by detection of the virus by PCR analysis of cord blood or neonatal blood collected within the first 12 h of birth. A probable case is defined as detection of the virus by PCR analysis in amniotic fluid collected prior to rupture of the membrane [13].

There is concern over vertical transmission due to the known tissue tropism and infection patho-mechanism of SARS-CoV-2 [14]. In Huang et al., the presence of plasma viral RNA was detected in 15% of patients [1], and therefore the possibility of the transmission of SARS-CoV-2 infection through blood is possible. The study by Valdéset al. [15] provides evidence of the uteroplacental location of Ang-(1-7) and the Angiotensin-Converting Enzyme 2 (ACE2) in both normal and pathological pregnancies. ACE2 expression is not only found in the syncytio-trophoblast and cytotrophoblast, but also in the endothelium and the vascular smooth muscle of the primary and secondary villi [15]. However, the analysis by Lü et al., [16] showed that a small proportion of the trophoblast cells expressed the ACE2 gene. This contrasts with evidence that receptors for the Zika virus and CMV, which cause congenital infections, are highly expressed by a variety of placental cell types [17]. On the other hand, foetal organs such as the heart and stomach (expression of angiotensin-converting enzyme inhibitors (ACE2), or adrenal glands and kidneys (co-expression of ACE-I and TMPRSS2) may be susceptible to SARS-CoV-2 infection [16]. Moreover, Jing et al. [18] reported the presence of ACE2 in the ovary, uterus, and vagina, which may result in infertility, menstrual disorders, foetal distress, and intrapartum transmission.

## 2. Materials and Methods

### 2.1. Study Design and Population

This prospective single-centre study was carried out in the Department of Obstetrics and Gynaecology, at the Central Clinical Hospital of the Ministry of the Interior and Administration in Warsaw, Poland. Between 15 May 2020 and 20 December 2020, a total of 91 pregnant women with COVID-19 infections were admitted for labour or with indication for induced labour. The COVID-19 diagnoses were confirmed either by positive antigen test or by positive RT-PCR assay performed within no more than 13 days prior to admission.

### 2.2. Study Procedures

To validate the pregnant women’s SARS-CoV-2 status, nasopharyngeal swabs were collected from each on the day of admission, and these produced 47 SARS-CoV-2 positive results. These pregnant women and the 48 infants delivered were included in the prospective observational study. We tested neonates twice: nasopharyngeal swabs were taken immediately after birth and either 24 h after (for isolated babies) or at discharge (when rooming-in). Whenever delivery was by caesarean section, a swab for SARS-CoV-2 rRT-PCR testing was taken from the amniotic cavity following uterus incision. Gene amplification was performed via reverse transcriptase real time polymerase chain reaction (rRT-PCR) which targeted the *N, E*, and *RdPd* genes. The assay was performed using a SARS-CoV-2 nucleic acid detection kit (GeneProof SARS-CoV-2 PCR Kit; GeneProof a.s., Brno, Czech Republic) following the kit manufacturer’s protocols. Our study only used rapid antigen tests that are characterized by a sensitivity of ≥80% and a specificity of ≥97%, thus meeting the conditions for diagnostic purposes, namely Panbio™ COVID-19 AG Rapid Test Device (Abbott, Abbot Rapid Diagnostics Jena GmbH, Jena, Germany) and Bioeasy 2019-nCoV Ag Fluorescence (Shenzhen Bioeasy Biotechnology Co. Ltd., Shenzhen, China). All participating women gave their informed written consent prior to participation, and the research was approved by the Bioethics Committee of the Central Clinical Hospital of the Interior and Administration in Warsaw.

### 2.3. Management

Following the recommendations of the Polish Society of Gynaecologists and Obstetricians, pregnant women with COVID-19 infections during labour, except for situations where the infection was either advanced or dynamic, or the labour was coming to an end, underwent caesarean section. As new data emerged, premature delivery and caesarean section were indicated for patients with severe or critical symptoms (worsening dyspnoea, respiratory failure resulting in the need for mechanical ventilation, or multiorgan failure). Visitors were prohibited during labour and delivery. Emotional support and patient–visitor interactions used non-contact alternatives, and video-calls were encouraged. At time of delivery, infants born to women diagnosed with COVID-19 were identified and treated as suspected of being infected (tested for SARS-CoV-2, and immediately isolated from the mother and healthy infants). After the health condition of the mothers and their new-born children was carefully assessed by healthcare professionals, rooming-in options and breastfeeding conditions were discussed, and introduced whenever possible.

### 2.4. Clinical Course of Illness

Patients were classified according to the guidelines of the Polish Society of Epidemiologists and Infectious Disease Physicians [19] into 1 of 4 groups, based on the severity of symptoms and examination results: mild (asymptomatic, or with the presence of cough, fever, dyspnoea, fatigue, myalgia, headache, nausea, vomiting, or diarrhoea), moderate (with clinical and radiological features of lung involvement), severe (with respiratory failure), or critical (with ARDS, symptoms of shock, multi-organ failure, or loss of consciousness).

## 3. Results

During the study period, a total of 91 pregnant women in labour or with indications for induction of labour who were infected with SARS-CoV-2 were admitted to hospital. Each was tested to confirm their SARS-CoV-2 status using positive rRT-PCR nasopharyngeal swabs, and 47 patients had a confirmed COVID-19 infection diagnosis. These 47 pregnant women were enrolled into the study and gave birth to 48 infants. The maternal characteristics and foetal outcomes are outlined in Table 1 and Table 2. In cases of delivery by caesarean section (n-41) a swab for SARS-CoV-2 rRT-PCR testing was taken from the amniotic cavity and produced 39 samples. The characteristics of the pregnant women with COVID-19 who underwent caesarean section with amniotic fluid sampling are presented in Table 3. All amniotic cavity swabs, as well as all nasopharyngeal swabs, whether taken at birth, or 24 h after delivery (when babies were isolated), or at discharge (when rooming-in) produced negative results.

The mean age of patients was 31 ± 5.2 years. The mean gestational age at diagnosis and delivery was 38.7 ± 1.7 weeks, ranging from 34 to 42 weeks. Preterm delivery before 37 weeks took place in 5 (10.6%) cases. Most patients (n-41, 87.2%) delivered via caesarean section with emergency indications in 30 cases (63.8%). Forty-three (896%) of the new-borns were isolated from their mothers after birth and fed by formula throughout the first 1–2 weeks of life. Five (10.4%) mothers decided to have immediate bonding and breastfeed using appropriate preventive equipment. One neonate (2.1%) was admitted to the neonatal intensive care unit (NICU) with prematurity as indication. APGAR scores at 1 min were 9.29 ± 1.57 and at 5 min were 9.63 ± 0.9. Low birth weight <2500 g was identified in 6 neonates (12.5%). Furthermore, during the study, no COVID-19 disease or sepsis of new-borns were documented. Additionally, no cases of maternal and perinatal death were reported.

## 4. Discussion

In our study, in 52% of the study group with infection confirmed earlier, either by positive antigen test or by positive RT-PCR assay, COVID-19 infection was confirmed by rRT-PCR nasopharyngeal swab.

It is necessary to confirm the diagnosis of SARS-CoV-2 infection in pregnant women by performing a RT-PCR assay and, according to WHO recommendations, in ambulatory patients or less severe respiratory disease SARS-CoV-2 virus detection can be performed using nasopharyngeal or oropharyngeal swabs [20]. Despite the fact that some studies indicate false-negative results for COVID-19 in 30%–40% of cases [21,22], nasopharyngeal or oropharyngeal swabs for rRT-PCR testing are still considered the diagnostic gold standard.

In our study, 872% of patients delivered via caesarean section, in 63.8% cases as an emergency and in 36.2% cases as a planned indication.

The respective short- and long-term advantages and disadvantages of delivery by caesarean section and vaginal delivery are well known. Compared with vaginal delivery, caesarean section is associated with a reduced rate of urinary incontinence and pelvic organ prolapse, but this must be weighed against the associated increased risk of the unfavourable course of future pregnancies in the mother and long-term childhood outcomes for the new-born [23]. Early studies of COVID-19 indicated that vaginal delivery is appropriate in mild and asymptomatic cases and may be associated with a low risk of intrapartum SARS-CoV-2 transmission to the new-born [24]. During vaginal delivery, amniotic fluid, vaginal bleeding, and vaginal discharge may increase the potential of infection [25]. Delivery by caesarean section in pregnant women with COVID-19 shortens the time of exposure and seems to be more beneficial for both patients and medical staff. Therefore, caesarean section, which permits an improved ventilation process for many patients, was not only reserved for women with a severe course of illness [24], but COVID-19 status alone also became a common indication for caesarean delivery early in the pandemic, despite a lack of evidence for vertical transmission. A review of 36 articles published between December 2019 and April 2020 by Debrabandere et al., [26] revealed that 68.9% of pregnant women delivered via caesarean section, with COVID-19 status alone being the common indication. Later, a study by Walker et al., [27] showed that the rate of infection is no greater than when the baby is born vaginally, and caesareans should continue to be performed for the normal obstetric indications. Another study indicated that women with COVID-19 infection undergoing caesarean section may have been at higher risk of adverse outcomes and that caesarean section is independently associated with an increased risk of clinical deterioration [28]. Current studies have indicated that 48% of deliveries of women with COVID-19 are by caesarean section [29].

Our study did not demonstrate any probable or confirmed case of vertical transmission, possibly due to the small number of patients in the study group or to imperfect measurement methods.

Nevertheless, vertical transmission of SARS-CoV-2 is possible, though rare [30], and the impact of the SARS-CoV-2 infection on the fetus during gestation remains unclear. Viral genomes were detected in vaginal swabs, maternal and umbilical cord plasma, amniotic fluid, placenta biopsies, and milk [31]. In one study by Shanes et al., [32] 16 of 16 examined placentas from patients with SARS-CoV-2 infection revealed signs of abnormal maternal circulation including features of mal-perfusion and intervillous thrombi. Therefore, in cases of SARS-CoV-2 infection, high rates of infertility, sterility, fetal death, fetal grow restriction, fetal anomalies, premature birth, and neonatal mortality may be suspected [33]. Low rates of vertical transmission identified in the literature may be related to efficient defence mechanisms, and the subject needs further investigation.

In our study, preterm delivery took place in five (10.6%) of the 47 cases of COVID-19 infected pregnant women, confirmed by hospital nasopharyngeal swab. Admission to neonatal intensive care was required in one neonate (2.1%) case. APGAR scores at 1 min were 9.29 ± 1.57 and at 5 min, 9.63 ± 0.9. Low birth weight <2500 g was identified in six neonates (12.5%). No symptoms of COVID-19 disease or sepsis of new-borns were documented and no perinatal death were reported. These findings do not negatively deviate from the most recent meta-analysis by Jafari et al. [29], which indicated preterm labour in 25% of cases, neonatal intensive care unit admission in 43% of cases, a mean of 9 in the 1 min APGAR score, a mean of 10 in the 5 min APGAR score, LBW (<2500 g) in 25% of cases, neonatal death in 2.5%, and stillbirth in 4%. Our study revealed a low rate of adverse perinatal outcomes that was probably due to safe neonatal management and our small patient group.

Following the guidelines of the Centres for Disease Control and Prevention, the Royal College of Obstetricians and Gynaecologists (RCOG), and the American College of Obstetricians and Gynaecologists (ACOG), family births are, under specific circumstances encouraged [34,35,36]. Because of the physical characteristics of our hospital, and to ensure the safety of patients and personnel, we did not permit visits to any of the labour, delivery, or hospitalization rooms. 89.6% of new-borns were immediately isolated from their mothers, with no skin-to-skin contact, and were bonded after two consecutive nasopharyngeal swabs (infant) with negative results and/or at the end of the mother’s 10–14-day isolation. Isolated infants were formula fed throughout the first 1–2 weeks of life. We also provided lactation support to encourage expression and storage of milk. After discussion with paediatricians, only 10.4% of the mothers felt comfortable with the potential risks of contact and chose rooming-in and breastfeeding. Mothers were informed about possible consequences of isolation including excessive stress, interruption of breastfeeding and reduced access to parent education about their new-born child. Although separation should be reserved for new-borns of women in the high-risk group, or when the mother is too ill to care for their infant, or when she needs higher levels of care [34,36], the lack of close contact between the baby and the mother decreases the likelihood of sharing infective respiratory droplets and causing horizontal infections [37]. The vertical transmission rate ranges from 3.2% [14] to 5.3% in recent studies [29]. The infection control measures we implemented resulted in no vertical transmission of SARS-CoV-2 and therefore findings of our study confirm previous reports. Similar approaches in the literature are likewise shown to have resulted in no perinatal transmission [38,39]. We believe that discussion about future directions for patient management to minimize perinatal viral transmission is still required and that adopting a strict policy even with asymptomatic patients may be essential due to new virus mutations [40]. In a study by Korber et al. [41], a SARS-CoV-2 variant that is currently spreading worldwide, carrying the Spike protein amino acid change D614G, is associated with lower RT-PCR cycle thresholds, suggesting higher upper respiratory tract viral loads. It might be speculated that increased levels of viral copies in maternal samples correlate with viral passage probability and, therefore, vertical, and horizontal transmission rates. Epidemiologists have concluded that the new SARS-CoV-2 lineage B.1.1.7, which is widespread in the UK, is more transmissible, with studies showing it can increase the number of new cases caused by an infected individual (Rt) by 40%–80% [42] and 50%–74% [43]. The other two variants, B.1.351 which is also known as South African mutation, and P1 (the Brazilian mutation) rapidly increase within local populations and their higher transmissibility has been confirmed [44,45]. As more alarming data about the characteristics of the new SARS-CoV-2 variants emerge [46,47,48,49], and as we witness the natural evolution and increased infection rates of the COVID-19 pandemic, different approaches to delivery and clinical management strategies for pregnant women and their new-born children need to be considered, and more research on viral transmission needs to be conducted.

## Figures and Tables

**Table 1:** Characteristics of COVID-19 patients in labour or indication for labour.

	Pregnant Women: SARS-CoV-2 RT-PCR Test Status of Nasopharyngeal Swab Performed on the Day of Admission, No. (%)		Infants: SARS-CoV-2 RT-PCR Test Status of Nasopharyngeal Swab Performed on the Day of Labour and after 24 h/on the Day of Discharge, No. (%)	
	Positive (*n* = 47)	Negative (*n* = 44)	Positive (*n* = 0)	Negative (*n* = 92)
Maternal age, mean ± SD, y	31 ± 5.2	32.2 ± 5.1	0 ± 0	31.6 ± 5.2
18–20	1 (2.1%)	0 (0.0%)	0 (0%)	1 (1.1%)
20–24	4 (8.5%)	2 (4.5%)	0 (0%)	6 (6.5%)
25–29	11 (23.4%)	10 (22.7%)	0 (0%)	22 (23.9%)
30–34	21 (44.7%)	18 (40.9%)	0 (0%)	40 (43.5%)
35–39	7 (14.9%)	11 (25.0%)	0 (0%)	18 (19.6%)
40–44	3 (6.4%)	3 (6.8%)	0 (0%)	6 (6.5%)
Week of pregnancy, median (range)	39 (34–41)	39 (33–41)	0 ± 0	39 (33–41)
31–33	0 (0.0%)	1 (2.3%)	0 (0%)	1 (1.1%)
34–36	5 (10.6%)	5 (11.7%)	0 (0%)	11 (11.9%)
37–39	25 (53.2%)	23 (52.3%)	0 (0%)	48 (52.2%)
40–42	17 (36.2%)	15 (34.1%)	0 (0%)	32 (34.8%)
SARS-CoV-2 RT-PCR test performed within no more than 13 days prior to operation, (*n* = 68), No. (%)	43 (91.5%)	39 (88.6%)	0 (0%)	83 (90.2%)
SARS-CoV-2 antigen test performed within no more than 13 days prior to operation, (*n* = 4), No. (%)	4 (8.5%)	5 (11.4%)	0 (0%)	9 (9.8%)
BMI, Mean ± SD	29.53 ± 5.29	29.65 ± 4.24	0 ± 0	29.58 ± 4.81
Comorbidities, No. (%)				
Diabetes	10 (21.3%)	7 (15.9%)	0 (0%)	17 (18.5%)
Hypertension	8 (17.0%)	7 (15.9%)	0 (0%)	15 (16.3%)
Hypothyroidism	15 (31.9%)	13 (29.5%)	0 (0%)	28 (30.4%)
Asthma	2 (4.3%)	1 (2.3%)	0 (0%)	3 (3.3%)
Cholestasis	1 (2.1%)	2 (4.5%)	0 (0%)	3 (3.3%)
Lupus erythematosus	2 (4.3%)	0 (0%)	0 (0%)	2 (2.2%)
Course of illness, No. (%)				
Mild	39 (82.9%)	43 (97.7%)	0 (0%)	83 (90.2%)
Moderate	7 (14.9%)	1 (2.3%)	0 (0%)	8 (8.7%)
Severe	1 (2.1%)	0 (0%)	0 (0%)	1 (1.1%)
Critical	0 (0%)	0 (0%)	0 (0%)	0 (0%)
Indication for caesarean section, No. (%)				
Emergency	30 (63.8%)	32 (72.7%)	0 (0%)	63 (68.5%)
Planned	11 (23.4%)	2 (4.5%)	0 (0%)	13 (14.1%)
Vaginal birth, No. (%)	6 (12.8%)	10 (22.7%)	0 (0%)	16 (17.4%)

**Table 2:** Infants’ characteristics.

	Pregnant Women: SARS-CoV-2 RT-PCR Test Status of Nasopharyngeal Swab Performed on the Day of Admission, No. (%)		Infants: SARS-CoV-2 RT-PCR Test Status of Nasopharyngeal Swab Performed on the Day of Labor and after 24 h/on the Day of Discharge, No. (%)	
	Positive (*n* = 48)	Negative (*n* = 44)	Positive (*n* = 0)	Negative (*n* = 92)
Preterm delivery, No. (%)	5 (10.4%)	5 (11.4%)	0 (0%)	11 (11.9%)
APGAR score, median (range)				
1 min	10 (2–10)	10 (8–10)	-	10 (2–10)
5 min	10 (5–10)	10 (8–10)	-	10 (5–10)
Low Birth Weight < 2500 g, No. (%)	6 (12.5%)	2 (4.5%)	0 (0%)	8 (8.7%)
NICU admission, No. (%)	1 (2.1%)	3 (6.8%)	0 (0%)	4 (4.3%)
Infant COVID-19 disease/perinatal death, No. (%)	0 (0%)	0 (0%)	0 (0%)	0 (0%)
Infant management No. (%)				
Isolation	43 (89.6%)	33 (75%)	0 (0 %)	76 (82.6%)
Rooming-in and breastfeeding	5 (10.6%)	11 (25%)	0 (0 %)	16 (17.4%)

**Table 3:** Characteristics of COVID-19 patients undergoing caesarean section with amniotic fluid swab sampling.

	SARS-CoV-2 RT-PCR Test Status of Nasopharyngeal Swab Performed on the Day of Surgery, No. (%)		SARS-CoV-2 RT-PCR Test Status of Amniotic Fluid Swab Performed on the Day of Surgery, No. (%)	
	Positive (*n* = 39)	Negative (*n* = 33)	Positive (*n* = 0)	Negative (*n* = 72)
Maternal age, mean ± SD, y	30.8 ± 5.4	32.5 ± 4.6	0 ± 0	31.6 ± 5.1
18–20	1 (2.6%)	0 (0.0%)	0 (0%)	1 (1.4%)
20–24	3 (7.7%)	0 (0.0%)	0 (0%)	3 (4.7%)
25–29	11 (28.2%)	8 (24.2%)	0 (0%)	19 (26.9%)
30–34	16 (41.0%)	15 (45.5%)	0 (0%)	31 (43.1%)
35–39	6 (15.4%)	8 (24.2%)	0 (0%)	13 (18.1%)
40–44	2 (5.1%)	2 (6.1%)	0 (0%)	4 (5.6%)
Week of pregnancy, median (range)	39 (34–41)	39 (33–41)	-	39 (33–41)
31–33	0 (0.0%)	1 (3.0%)	0 (0%)	1 (1.4%)
34–36	4 (10.3%)	5 (15.2%)	0 (0%)	9 (12.5%)
37–39	23 (58.9%)	18 (54.5%)	0 (0%)	41 (56.9%)
40–42	12 (30.8%)	9 (27.3%)	0 (0%)	21 (29.2%)
SARS-CoV-2 RT-PCR test performed within no more than 13 days prior to operation (*n* = 68), No. (%)	36 (92.3%)	32 (96.9%)	0 (0%)	68 (94.4%)
SARS-CoV-2 antigen test performed within no more than 13 days prior to operation (*n* = 4), No. (%)	3 (7.7%)	1 (3.1%)	0 (%)	4 (5.6%)
BMI, Mean ± SD	29.97 ± 4.29	29.46 ± 5.4	0 ± 0	29.69 ± 4.91
Comorbidities, No. (%)				
Diabetes	7 (17.9%)	7 (21.2%)	0 (0%)	14 (19.4%)
Hypertension	7 (17.9%)	7 (21.2%)	0 (0%)	13 (18.1%)
Hypothyroidism	12 (30.8%)	8 (24.2%)	0 (0%)	20 (27.8%)
Asthma	2 (5.1%)	1 (3.0%)	0 (0%)	3 (4.2%)
Cholestasis	1 (2.6%)	2 (6.1%)	0 (0%)	3 (4.2%)
Lupus erythematosus	2 (5.1%)	0 (0%)	0 (0%)	2 (2.8%)
Course of illness, No. (%)				
Mild	31 (79.5%)	32 (96.9%)	0 (0%)	63 (87.5%)
Moderate	7 (17.9%)	1 (3.1%)	0 (0%)	8 (11.1%)
Severe	1 (2.6%)	0 (0%)	0 (0%)	1 (1.4%)
Critical	0 (0%)	0 (0%)	0 (0%)	0 (0%)
Indication for caesarean section, No. (%)				
Emergency	28 (71.8%)	31 (93.9%)	0 (0%)	59 (81.9%)
Planned	11 (28.2%)	2 (6.1%)	0 (0%)	13 (18.1%)

## Data Availability

The datasets generated and/or analyzed during the current study are not publicly available due to our policy but are available from the corresponding author upon reasonable request.
